# Supplementary material for: Leukoaraiosis as a Predictor of Depression and Cognitive Impairment among Stroke Survivors: A Systematic Review
Source: Neurol Int. 2023 Feb 13;15(1):238–72. doi: 10.3390/neurolint15010016 (PMC9944578; doi:10.3390/neurolint15010016)
Supplement: Supplementary file 1 [file neurolint-15-00016-s001.zip › neurolint-2160049-supplementary-done.pdf]

neurology international

MDPI

Systematic Review

Leukoaraiosis as a Predictor of Depression and Cognitive Impairment among Stroke Survivors: A Systematic Review

Eftychia Tziaka [1,†], Foteini Christidi [1,†], Dimitrios Tsiptsios [1,*], Anastasia Sousanidou [1], Stella Karatzetzou [1], Anna Tsiakiri [1], Triantafyllos K. Doskas [1], Konstantinos Tsamakis [2], Nikolaos Retzepis [3], Christos Konstantinidis [3], Christos Kokkotis [3], Aspasia Serdari [4], Nikolaos Aggelousis [3] and Konstantinos Vadikolias [1]

1 Neurology Department, Democritus University of Thrace, 68100 Alexandroupolis, Greece
2 Institute of Psychiatry, Psychology and Neuroscience (IoPPN), King's College London, London SE5 8AB, UK
3 Department of Physical Education and Sport Science, Democritus University of Thrace, 69100 Komotini, Greece
4 Department of Child and Adolescent Psychiatry, Medical School, Democritus University of Thrace, 68100 Alexandroupolis, Greece
* Correspondence: tsiptsios.dimitrios@yahoo.gr; Tel.: +30-6944320016
† These authors contributed equally to this work.

Citation: Tziaka, E.; Christidi, F.; Tsiptsios, D.; Sousanidou, A.; Karatzetzou, S.; Tsiakiri, A.; Doskas, T.K.; Tsamakis, K.; Retzepis, N.; Konstantinidis, C.; et al. Leukoaraiosis as a Predictor of Depression and Cognitive Impairment among Stroke Survivors: A Systematic Review. *Neurol. Int.* **2023**, *15*, 238–272. https://doi.org/10.3390/neurolint15010016

Academic Editor: Tibor Hortobágyi

Received: 29 December 2022
Revised: 2 February 2023
Accepted: 6 February 2023
Published: 13 February 2023

Copyright: © 2023 by the authors. Licensee MDPI, Basel, Switzerland. This article is an open access article distributed under the terms and conditions of the Creative Commons Attribution (CC BY) license (https://creativecommons.org/licenses/by/4.0/).

Supplementary Table 1. Characteristics of the included studies focusing on depression.

	1st Author (Year)	Cerebrovascular Risk Factors (n) / Previous stroke	mNOS (0-8) [Selection (3); Comparability (2); Outcome (3)]
1.	Guo (2022) [55]	• Hypertension n=246, Diabetes mellitus n=135, Smoking n=135<br>• Previous stroke n=0	Selection (3)<br>Comparability (2)<br>Outcome (2)<br><br>Total = 7
2.	Jaroonpipatkul (2022) [54]	• Smoking n=3, Hypertension n=11, Dyslipidemia n=12, Diabetes mellitus n=16, Ischemic heart disease n=6, Atrial fibrillation n=4<br>• Previous stroke n=9	Selection (3)<br>Comparability (2)<br>Outcome (3)<br><br>Total = 8
3.	Zhou (2022) [56]	• Hypertension n=241, Diabetes mellitus n=80, Hyperlipidemia n=41, Atrial fibrillation n=33, Coronary heart disease n=6, Smoking n=90, Alcohol n=62<br>• Previous stroke n=44	Selection (3)<br>Comparability (2)<br>Outcome (2)<br><br>Total = 7
4.	Douven (2020) [44]	• n/a<br>• n/a	Selection (3)<br>Comparability (2)<br>Outcome (3)<br><br>Total = 8
5.	Bae (2019) [51]	• Hypertension n=204, Diabetes mellitus n=112, Heart disease n=33, Hypercholesterolemia n=229<br>• Previous stroke n=34	Selection (3)<br>Comparability (2)<br>Outcome (3)<br><br>Total = 8
6.	Carnes-Vendrell (2019) [58]	• Alcohol n=38, Smoking n=23, Diabetes mellitus n=30, Hypertension n=56, Hypercholesterolemia n=37, Atrial fibrillation n=5<br>• Previous stroke n=14	Selection (2)<br>Comparability (2)<br>Outcome (3)<br><br>Total = 7
7.	Pavlovic (2016) [59]	• Hypertension n=264, Diabetes mellitus n=76, Hypercholesterolemia n=245, Smoking n=99, Coronary artery disease n=46, Peripheral artery disease n=19, atrial fibrillation n=13, Carotid stenosis >50% n=20<br>• Previous stroke n/a	Selection (3)<br>Comparability (2)<br>Outcome (3)<br><br>Total = 8
8.	Tanislav (2015) [46]	• Hypertension n=961, Diabetes mellitus n=195, Hyperlipidemia n=637, Obesity n=459, Tobacco smoking n=1103, Cardiovascular disease n=165, Physical inactivity n=924, Obstructive sleep apnea n=72, Sleep ≤6 h at night	Selection (2)<br>Comparability (1)<br>Outcome (2)<br><br>Total = 5

time n=367, Regular alcohol consumption n=1017, Higher alcohol consumption n=650<br>• Previous stroke n=0

Notes. n/a = not available; n = number; mNOS = modified Newcastle-Ottawa Scale.

Supplementary Table 2. Characteristics of included studies focusing on cognition.

	1st Author (Year)	Cerebrovascular Risk Factors (n) / Previous stroke	mNOS (0-8) [Selection (3); Comparability (2); Outcome (3)]
1.	Georgakis (2022) [68]	• Hypertension n=515, Diabetes mellitus n=131, Smoking n=155, Alcohol n=498, Atrial fibrillation n=133<br>Previous stroke n=71	Selection (3)<br>Comparability (2)<br>Outcome (3)<br><br>Total = 8
2.	Fruhwirth (2021) [100]	• Hyperlipidemia n=65, Hypertension n=61, Active smoking n=32, Diabetes mellitus n=12, Obesity n=10<br>• Previous stroke n/a	Selection (3)<br>Comparability (2)<br>Outcome (3)<br><br>Total = 8
3.	Pasi (2021) [98]	• Smoking n=66, Alcohol n=321, Hypertension n=458, Diabetes n=121, coronary artery disease n=101, Atrial fibrillation n=99<br>• Previous stroke n=75	Selection (3)<br>Comparability (2)<br>Outcome (3)<br><br>Total = 8
4.	Peng (2021) [79]	• Hypertension n=112, Smoking n=34, Diabetes mellitus n=48, Coronary heart disease n=32, Atrial fibrillation n=31<br>• Previous stroke n/a	Selection (2)<br>Comparability (1)<br>Outcome (2)<br><br>Total = 5
5.	Sung (2021) [85]	• Hypertension n=90, Diabetes mellitus n=40, Hyperlipidemia n=88, Atrial fibrillation n=9<br>• Previous stroke n=0	Selection (3)<br>Comparability (2)<br>Outcome (3)<br><br>Total = 8
6.	Appleton (2020) [67]	• Hypertension n=2607, Diabetes mellitus n=699, Atrial fibrillation n=762, Ischemic heart disease n=669, Peripheral artery disease n=117, Hyperlipidemia n=1098, Smoking n=945, Alcohol n=294<br>• Previous stroke: 1138	Selection (2)<br>Comparability (1)<br>Outcome (3)<br><br>Total = 6
7.	Suda (2020) [80]	• Smoking n=40, Alcohol n=63, Hypertension n=78, Diabetes mellitus n=34, Dyslipidemia n=50, Ischemic heart disease n=7, Atrial fibrillation n=10<br>• Previous stroke n=17	Selection (3)<br>Comparability (2)<br>Outcome (2)<br><br>Total = 7
8.	Yatawara (2020) [87] *	• n/a<br>• n/a	Selection (3)<br>Comparability (2)<br>Outcome (2)<br><br>Total = 7

9.	Du (2019) [69]	• Hypercholesterolemia n=13, Diabetes mellitus n=49, Hypertension n=90, Smoking n=57<br>• Previous stroke n/a	Selection (3)<br>Comparability (2)<br>Outcome (1)<br><br>Total = 6
10..	Molad (2019) [84]	• Smoking n=89, Diabetes mellitus n=109, Dyslipidemia n=211, Hypertension n=232<br>• Previous stroke n=0	Selection (2)<br>Comparability (2)<br>Outcome (3)<br><br>Total = 7
11.	Liang (2019) [78]	• Current or previous smoking n=157, Hypertension n=292, Hyperlipidemia n=184, Diabetes mellitus n=114, Ischemic heart disease n=19, Atrial fibrillation n=17<br>• Previous stroke n=0	Selection (3)<br>Comparability (2)<br>Outcome (3)<br><br>Total = 7
12.	Zamboni (2019) [89]	• Hypertension n=260, Diabetes mellitus n=55, Atrial fibrillation n=60, Hyperlipidemia n=163, Smoking n=159<br>• n/a	Selection (2)<br>Comparability (2)<br>Outcome (2)<br><br>Total = 6
13.	Hawe (2018) [65]	• n/a<br>• Previous stroke n=0	Selection (2)<br>Comparability (1)<br>Outcome (3)<br><br>Total = 6
14.	Puy (2018) [66]	• Hypertension n=206, Diabetes mellitus n=70, Hypercholesterolemia n=152, Smoking n=81, Alcohol n=15<br>• Previous stroke n=26	Selection (3)<br>Comparability (2)<br>Outcome (3)<br><br>Total = 8
15.	Yatawara (2018) [86] *	• n/a<br>• n/a	Selection (3)<br>Comparability (2)<br>Outcome (3)<br><br>Total = 8
16.	Divya (2017) [94]	• Diabetes mellitus n=28, Hypertension n=44, Dyslipidemia n=24, Smoking n=18<br>• Previous stroke n/a	Selection (2)<br>Comparability (2)<br>Outcome (3)<br><br>Total = 7
17.	Cao (2017) [77]	• Hypertension n=44, Diabetes mellitus n=14, Hypercholesterolemia n=9, Smoking n=15<br>• Previous stroke n/a	Selection (2)<br>Comparability (2)<br>Outcome (2)<br><br>Total = 6
18.	Molad (2017) [95]	• Hypertension n=149, Diabetes mellitus n=64, Dyslipidemia n=138, Ischemic heart disease n=30, Smoking n=57<br>• Previous stroke n=0	Selection (2)<br>Comparability (2)<br>Outcome (3)<br><br>Total = 7

19.	Sivakumar (2017) [93]	• Hypertesion n=69, Diabetes mellitus n=21, Dyslipidemia n=57<br>• Previous stroke n=29	Selection (2)<br>Comparability (2)<br>Outcome (3)<br><br>Total = 7
20.	Zhang (2017) [81]	• Hypertension n=156, Diabetes n=111, Dyslipidemia n=53, Coronary artery disease n=13, Smoking n=90<br>• Previous stroke n=0	Selection (3)<br>Comparability (1)<br>Outcome (2)<br><br>Total = 6
21.	Mandzia (2016) [92]	• Hypertension n=79, Diabetes mellitus n=16, Previous myoardial infarction n=11, Smoking n=13, Atrial fibrillation n=8, Obstructive sleep apnea n=67<br>• Previous stroke n/a	Selection (3)<br>Comparability (2)<br>Outcome (3)<br><br>Total = 8
22.	Moulin (2016) [97] **	• Ischemic heart attack n=21, alcohol 65, Hypertension n=141, Diabetes n=31, Hypercholestrolemia n=69, Smoking n=42<br>• Previous stroke n=33	Selection (3)<br>Comparability (2)<br>Outcome (3)<br><br>Total = 8
23.	Benedictus (2015) [97] **	• Hypertension n=105, Diabetes mellitus n=23, Hypercholesterolemia n= 51, Smoking n=32, alcohol n=54, atrial firbrillation n=18<br>• Previous stroke n=25	Selection (3)<br>Comparability (2)<br>Outcome (3)<br><br>Total = 8
24.	Kumral (2015) [62]	• Hypertension n=7388, Current cigarette smoker n=1678, Diabetes mellitus n=2810, Coronary heart disease n=2015, Atrial fibrillation n=1645, Hyperhomocysteinemia n=1175, Hypercholesterolemia n=3481, Hypertriglyceridemia n=2240, Higher LDL cholesterol n=4339, Lower HDL cholesterol n=5755<br>• Previous stroke n/a	Selection (2)<br>Comparability (1)<br>Outcome (3)<br><br>Total = 6
25.	Nakano (2015) [71]	• Hypertension n=91, Dyslipidemia n=66, Diabetes 3 n=5, Atrial fibrillation n=18, Ischemic heart disease n=12<br>• Previous stroke n/a	Selection (2)<br>Comparability (2)<br>Outcome (3)<br><br>Total = 7

Notes. Studies from the same group or cohort are highligted with * and **. n/a = not available; n = number; mNOS = modified Newcastle-Ottawa Scale.

Supplementary Table 3. Characteristics of included studies focusing on depression and cognition.

	1st Author (Year)	Cerebrovascular Risk Factors (n) / Previous stroke	mNOS (0-8) [Selection (3); Comparability (2); Outcome (3)]
1.	Douven (2018) [42]	• n/a<br>• n/a	Selection (3)<br>Comparability (2)<br>Outcome (3)<br><br>Total = 8

Notes. n/a = not available; n = number; mNOS = modified Newcastle-Ottawa Scale
